# The Tyrosine Kinase Inhibitor Dasatinib Induces a Marked Adipogenic Differentiation of Human Multipotent Mesenchymal Stromal Cells

**DOI:** 10.1371/journal.pone.0028555

**Published:** 2011-12-02

**Authors:** Adriana Borriello, Ilaria Caldarelli, Maria Assunta Basile, Debora Bencivenga, Annunziata Tramontano, Silverio Perrotta, Fulvio Della Ragione, Adriana Oliva

**Affiliations:** 1 Department of Biochemistry and Biophysics, Second University of Naples, Naples, Italy; 2 Department of Pediatrics, Second University of Naples, Naples, Italy; Virginia Commonwealth University, United States of America

## Abstract

**Background:**

The introduction of specific BCR-ABL inhibitors in chronic myelogenous leukemia therapy has entirely mutated the prognosis of this hematologic cancer from being a fatal disorder to becoming a chronic disease. Due to the probable long lasting treatment with tyrosine-kinase inhibitors (TKIs), the knowledge of their effects on normal cells is of pivotal importance.

**Design and Methods:**

We investigated the effects of dasatinib treatment on human bone marrow-derived mesenchymal stromal cells (MSCs).

**Results:**

Our findings demonstrate, for the first time, that dasatinib induces MSCs adipocytic differentiation. Particularly, when the TKI is added to the medium inducing osteogenic differentiation, a high MSCs percentage acquires adipocytic morphology and overexpresses adipocytic specific genes, including *PPARγ, CEBPα, LPL* and *SREBP1c*. Dasatinib also inhibits the activity of alkaline phosphatase, an osteogenic marker, and remarkably reduces matrix mineralization. The increase of PPARγ is also confirmed at protein level. The component of osteogenic medium required for dasatinib-induced adipogenesis is dexamethasone. Intriguingly, the increase of adipocytic markers is also observed in MSCs treated with dasatinib alone. The TKI effect is phenotype-specific, since fibroblasts do not undergo adipocytic differentiation or PPARγ increase.

**Conclusions:**

Our data demonstrate that dasatinib treatment affects bone marrow MSCs commitment and suggest that TKIs therapy might modify normal phenotypes with potential significant negative consequences.

## Introduction

The treatment of chronic myelogenous leukemia (CML) with specific tyrosine kinase inhibitors (TKIs) has entirely mutated the prognosis of this hematologic cancer from being a fatal disorder to becoming a chronic disease, in which the patients might be on treatment for decades [1]–[4]. Three major TKIs are employed in CML therapy, namely imatinib mesylate (ST-571, Gleevec®, Novartis), nilotinib (AMN107, Tasigna®, Novartis), and dasatinib (BMS354825, Sprycel®, Bristol Myers Squibb).

Imatinib mesylate was introduced in therapy in 1998 as a potent and specific inhibitor of BCR-ABL protein, the recombinant kinase responsible for CML development [5]. The molecule is also able to inhibit the tyrosine kinase activity of ABL, c-Kit, c-FMS, Syk and the receptor of platelet-derived growth factor (PDGFR) [3]. Imatinib is highly effective in chronic phase CML [6]. The TKI induces a high percentage of complete hematologic response, and a significant progression-free survival rate without accelerated or blast crisis [7], [8].

However, a small number of patients develop a suboptimal response or resistance to imatinib mesylate [9]. In order to overcome this resistance, second-generation BCR-ABL inhibitors have been developed, including nilotinib and dasatinib. Nilotinib, although more potent than imatinib in inhibiting BCR-ABL activity, displays a selectivity for the same target kinases as imatinib [10], [11]. No significant activity is shown against other kinases including Src, FLT3, VEGFR, EGFR, InsR, RET, MET or IGFR [12]. Dasatinib is a powerful, second-generation, multitarget kinase inhibitor of BCR-ABL. The compound was developed initially as an inhibitor of Src family kinases such as Fyn, Yes, Src, and Lyk, but it also inhibits BCR-ABL, EphA2, PDGFR, and c-Kit [13], [14]. Additionally, it binds to and inhibits other tyrosine and serine/threonine kinases, such as mitogen-activated protein kinases (MAPK) and tyrosine kinase discoidin domain receptor [13], [14].

Patients affected by CML and treated with imatinib have been reported to show alteration of bone metabolism. A number of studies suggested initially that the TKI might have a positive effect on bone mass [15]–[17]. Particularly, O'Sullivan and colleagues observed a 4% increase in spinal bone mass density in nine imatinib-treated CML patients, not paying, however, attention to the subjects body weight and fat mass increase [15] that are factors known to positively influence bone mass [18].

Very recently, the same group has demonstrated that in healthy rodents imatinib treatment does not increase bone mass [19]. They have also suggested that the previously reported improvements in bone density of patients receiving imatinib may not be directly dependent on the drug [19]. Indeed, either a decrease of serum osteocalcin or an osteoblast number reduction have been consistently reported, being coherent with imatinib's inhibition of osteoblast proliferation and survival *in vitro* [20]. These findings also agree with additional data from human studies reporting a lowering of osteogenesis serum markers after long-term imatinib therapy [15], [17], [21], [22]. In summary, the majority of data, although not being conclusive, suggest that imatinib impairs osteoblast proliferation and, after a prolonged therapy, negatively affect bone turn-over.

Bone marrow is a complex cellular array containing stem cells of both hematopoietic and nonhematopoietic lineages. Hematopoietic stem cells are capable of self-renewal and can develop into any kind of blood cell, while the nonhematopoietic elements, called mesenchymal stem cells or, better, mesenchymal stromal cells (MSCs) [23], create a supportive bone marrow niche for the hematopoietic stem cells [24], [25]. In addition, MSCs can differentiate into a variety of cell phenotypes including osteoblasts, adipocytes and chondrocytes [26]–[29]. The two types of bone marrow stem cells interact and dialogue each other and a series of studies has suggested the importance of cytokine production and reciprocal contact for the growth and survival of both bone marrow cellular components [28]. Thus, MSCs have a pivotal role in bone remodeling and play a central function in haematopoiesis.

Here we investigated the effect of dasatinib on human MSCs differentiation. The interest in this study stems from the MSCs multiple roles and the probable prolonged course of dasatinib treatment. Moreover, the multitarget kinase inhibitor was selected since it has been approved for use in CML patients and is under evaluation for the treatment of numerous other cancers.

## Materials and Methods

### Ethics statements

K562 cell line strain employed in this study was kindly given by Prof. P. Auberger, INSERM U895 (C3M), Faculté de Medecine, Nice, FRANCE. MSCs were obtained employing heparinized human bone marrow from healthy volunteers. The study was approved by the Medical Ethics Committee of the Second University of Naples and was performed in accordance with the Declaration of Helsinki. Written informed consent was obtained from all subjects prior to participation in the study.

## Reagents

Dasatinib and bosutinib were furnished by LC Laboratories (Woburn, MA, USA). Tissue culture biochemicals were from Invitrogen (Grand Island, NY, USA), and plasticware was obtained from BD Falcon (Franklin Lakes, NJ, USA). All the chemicals were obtained, unless otherwise stated, from Sigma (St Louis, MO, USA), and were of the highest grade commercially available. The nuclear and cytoplasmic extraction kit (Ne-Per) was furnished by Pierce Biotechnology (Rockford, IL, USA). Monoclonal antibodies against PPARγ (E8, sc-7273), phospho-Erk1/2 (K23, sc-7383), phosphotyrosine (PY20, sc-508), and polyclonal antisera against Erk1/2 (K23, sc-94), HDAC1 (H51m, sc-7872), cofillin (FL-166 sc-33779) were from Santa Cruz Biotechnology (Santa Cruz, CA, USA). Monoclonal antibodies against p27^Kip1^ were from Transduction Laboratories (Lexington, UK). Polyclonal antibodies against β-actin were from Sigma.

### Preparation and characterization of human bone marrow-derived mesenchymal stromal cells

MSCs were obtained employing heparinized human bone marrow from healthy volunteers. MSCs cultures were initiated as previously described [30]. Briefly, the bone marrow sample was diluted 1:5 with Opti-MEM containing 10% fetal calf serum (FCS), penicillin 100 units/ml, streptomycin 100 µg/ml and sodium ascorbate 50 µg/ml (growth medium) and incubated at 37°C in a 5% CO_2_ humidified atmosphere. After 48 h incubation, the medium was collected and centrifuged at 800xg. The pellet was discarded, while the corresponding supernatant was added again to the dish. In three-four days, several foci of adherent spindle-like cells appeared and grew until subconfluence in the following two weeks. Afterwards, cells were trypsinized, and their phenotype was confirmed by flow cytometry using a wide panel of labeled monoclonal antibodies. Precisely, MSCs expressed specific surface markers, such as CD13 (clone L138, BD Biosciences, Milan, Italy), CD29 (Anti-Integrin beta 1 antibody, clone EP1041Y-Carboxyterminal end, Abcam, Cambridge, USA), CD44 (Anti-CD44 antibody, clone F10-44-2, Abcam), CD105 (clone SN6; Serotec, Kidlington, Oxford, UK), and were negative for hematopoietic cell markers CD14 (clone MFP9, BD Biosciences), CD34 (clone 581, BD Biosciences), and CD45 (clone HI30, BD Biosciences) [30]. Analysis of cell populations was done by means of direct immunofluorescence with a FACSCalibur flow cytometer (BD Biosciences) and data were calculated using CellQuest software (BD Biosciences). Figure S1 reports an example of MCSs FACS analysis. Only early cultures, between the second and fourth passage, were used in our experiments.

### Induction of osteoblastic differentiation and staining methods

The effects of the tested TKI on osteogenic differentiation were evaluated analyzing the expression of two specific markers, namely alkaline phosphatase (AP) activity and mineralization of the extracellular matrix. Usually, cells were seeded at a density of 10^4^ cells/cm^2^ in 6-well plates. The day after, the medium was replaced by fresh medium supplemented with or without the molecules under investigation. After 7 days, AP specific activity was evaluated. Once removed the medium, wells were rinsed with 20 mM Tris/HCl, pH 7.4, 0.5 M NaCl (TBS) and the cells lysed by a specific buffer (20 mM Tris/HCl, pH 7.4, 0.5 mM NaCl, 0.25% Triton X-100, 0.5 mM phenylmethylsulfonyl fluoride, 0.5 mM DTT). After 30 min in ice, the cell lysates were centrifuged at 13,000*g* for 5 min, and the supernatants assayed for AP activity. AP activity was determined by measuring the release of para-nitrophenol from disodium *para*-nitrophenyl phosphate. The reaction mixture contained 10 mM disodium *para*-nitrophenyl phosphate, 0.5 mM MgCl_2_, 0.1 M diethanolamine phosphate buffer pH 10.5, and 10**–**30 µg of cell lysate in a final volume of 0.5 ml. After 30 min at 37°C, the reaction was stopped by adding 0.5 ml of 0.5 M NaOH. *Para*-nitrophenol levels were measured spectrophotometrically at a wavelength of 405 nm. One unit was defined as the amount of enzyme that hydrolyzes 1 nmol of *para*-nitrophenyl phosphate/min under the specified conditions.

For *in vitro* mineralization experiments, cells were plated at 10^4^ cells/cm^2^ in 6-well plates and, after a day, incubated in a medium supplemented with or without the molecules under investigation. After one week, the osteogenic medium (OM) was added (50 µg/ml L-ascorbic acid, 10 mM β-glycerophosphate and 100 nM dexamethasone) and the cells were cultured as specified in the Results Section.

Both the *o*-cresolphthalein complexone method and Alizarin Red Staining were employed to evaluate the mineralization of the extracellular matrix. For the *o*-cresolphthalein complexone method, the cells were washed with PBS, scraped from the plate, transferred into a 1.5-ml microcentrifuge tube and subjected to the decalcification with 0.6 N HCl for 24 h at room temperature. The slurry was then centrifuged at 12,000*g* for 10 min and 500 µl of the supernatant were transferred into a new tube: the calcium released in the supernatants was determined colorimetrically at 540 nm. For Alizarin Red Staining method, cells were washed with PBS and fixed in 10% (v/v) formaldehyde for 15 min at room temperature. The monolayers were washed with distilled water and added with 1 ml of 40 mM Alizarin Red S (pH 4.1). After 20 min incubation at room temperature with gentle shaking, the unincorporated dye was removed and the cells were washed with distilled water prior to proceed to the quantification. For quantification of staining, 800 µl 10% acetic acid was added to each well. The monolayers were then scraped from and transferred into a 1.5-ml microcentrifuge tube. After vortexing for 30 s, the slurry was overlaid with 500 µl mineral oil, heated to 85 °C for 10 min, transferred to ice for 5 min, and centrifuged at 20,000g for 15 min. Then, 500 µl of the supernatant was removed to a new tube and 200 µl of 10% ammonium hydroxide was added to neutralize the acid. Aliquots (150 µl) of the supernatant were read in triplicate at 405 nm.

### Induction of adipocytic differentiation and Sudan red staining

Cells were seeded at 10^4^ cells/cm^2^ and cultured in the standard growth medium. At 90**–**100% cell confluence, the medium was supplemented with the adipogenic components (1 µM dexamethasone, 0.45 mM 3-isobutyl-1-methylxanthine, 6 µM insulin), and MSCs were cultured as described in Results Section. Adipocytic differentiation was evaluated by using Sudan red staining. Briefly, cells were fixed in 0.5% glutaraldehyde for 15 min at room temperature and rinsed twice in PBS. Filtered Sudan red supersaturated solution in 60% isopropanol was added to the MSCs: after 15 min staining, the cells were washed once with 60% isopropanol and twice with PBS. Adipocytes, containing lipid droplets, stained red.

### Semi-quantitative reverse transcriptase-PCR (RT-PCR) assay

Reverse-transcriptase PCR was mainly performed as reported [31]. The sequences of the primers employed for the different transcripts analyzed (PPARγ, CEBPα, LPL, SREBP1c and GAPDH) are available on request. After PCR reaction, the products were separated by electrophoresis on 2% (w/v) agarose gel and visualized by ethidium bromide staining. Gels were scanned with FLA-5000 imaging system (Fujifilm, Tokyo, Japan) and Image Reader software (Raytest®, Straubenhardt, Germany) [31].

### Quantitative RT- PCR

Messenger RNA expression of PPARγ was quantified by quantitative RT-PCR using SYBR® Green dye (SYBR® Green PCR Master Mix, Applied Biosystems, Foster City, CA, USA) and sequence-specific primers [31]. Total RNA from control and treated cells was isolated using Tripure method (Roche Diagnostics, Monza, Italy) [31]. Reverse transcription was performed using 1 µg total RNA and the reverse transcription system (Promega, Madison, WI, USA). The amplification was performed in an ABI PRISM® 7900 Sequence Detection System (Applied Biosystems, Monza, Italy) with 40 cycles of a two-step PCR (95°C for 15 sec and 60°C for 60 sec) after an initial activation step (95°C for 10 min). Melting curves from 60°C to 99°C were assessed to evaluate specificity. Serial dilutions of purified amplicons served to generate standard melting curves. Relative quantification was calculated by normalizing the test crossing thresholds (Ct) with the β-actin amplified control Ct. The sequences of PPARγ and β-actin primers employed in the quantitative RT- PCR are available on request.

### Cellular Fractionation and immunoblotting

Total cell extracts were prepared as reported [31], [32]. Nuclear and cytosol extracts were prepared using Ne-Per reagent, following manufacturer's instructions (Pierce). Extracts obtained by this method were preliminary tested for cross-contamination using antibodies against HDAC1 protein (as nuclear markers) and against cofillin (as cytosolic proteins). Immunoblotting analyses were carried out essentially as reported [31], [32].

## Results

### 1. Effect of dasatinib on the differentiation of MSC

**Figure 1:**
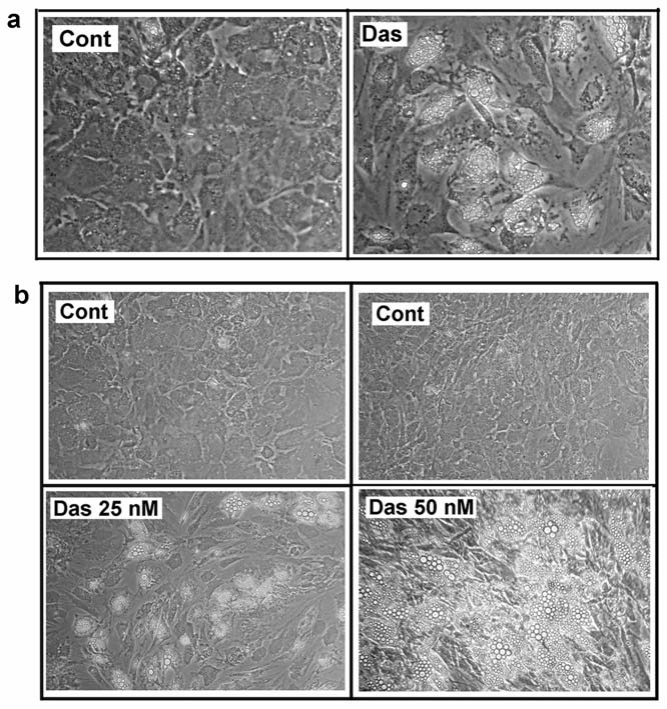
Effect of dasatinib on MSCs phenotype. **Panel a**) MSCs were seeded at a density of 10^4^ cells/cm^2^. After one day, 25 nM dasatinib (Das) was added. After one week, the osteogenic medium replaced the growth medium. The osteogenic medium containing the TKI was changed every 3 days. Finally, after additional 3 weeks, the images of MSCs were captured as described in Materials and Methods. **Panel b**) The experiments were performed exactly as reported in panel a, except that the dasatinib was employed at 25 and 50 nM. The figure is representative of 3 independent experiments.

In order to investigate the activity of dasatinib on the mineralization process, we tested the effect of the molecule on osteoblastic differentiation of bone-marrow human MSCs. To this aim, MSCs, plated at high density, were treated with dasatinib for 7 days. Afterwards, the cells were cultured in OM supplemented with the TKI. The dasatinib concentration employed (25 nM) was chosen on the basis of the expected plasma TKI level of CML-treated patients and the amount usually employed in studies with other cellular models. Surprisingly, after 3 weeks, we evidenced that the treatment not only negatively affected mineralization but also it induced morphological changes typical of the adipocytic differentiation (**Figure 1A**). In order to confirm the observation, we incubated MSCs with two different amounts of the TKI. As reported, in **Figure 1B**, adipogenesis was particularly evident after incubation with 50 nM dasatinib.

**Figure 2:**
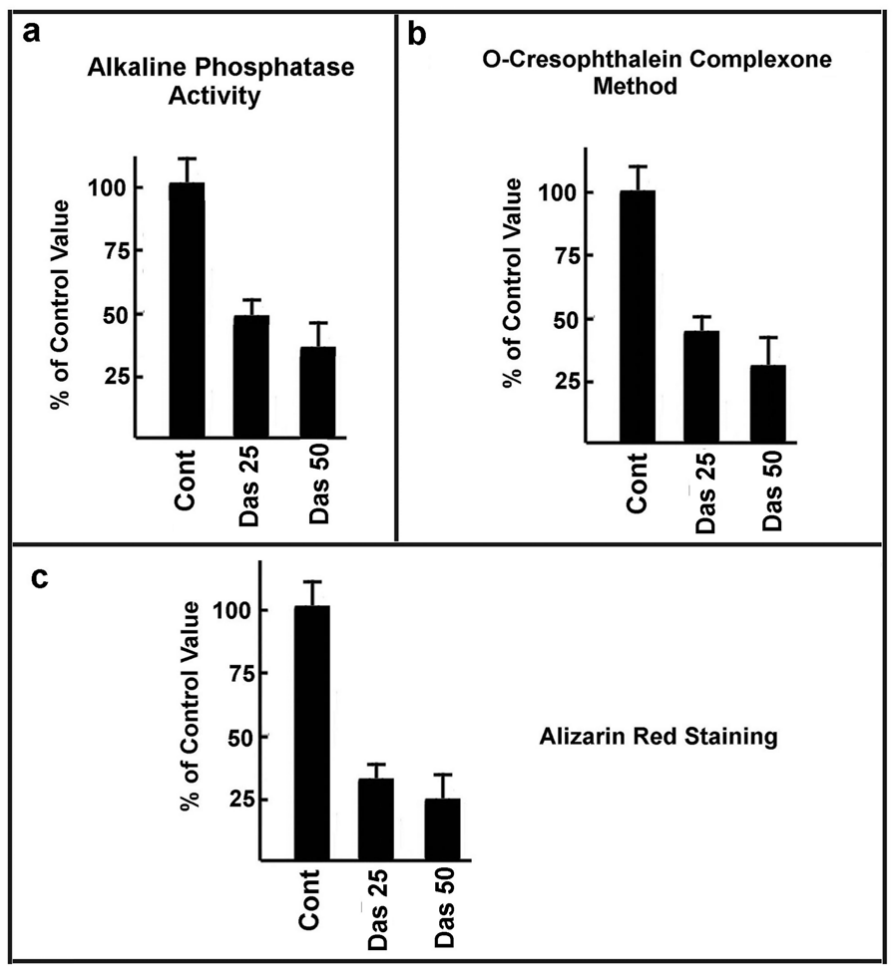
Characterization of dasatinib effect on MSCs osteogenesis. **Panel a**) MSCs cells were treated for one week with 25 nM or 50 nM dasatinib (Das). Then, the activity of alkaline phosphatase was evaluated as described in Materials and Methods and expressed as percentage of control. **Panel b**) MSCs cells were treated with 25 nM or 50 nM dasatinib (Das) following the scheme reported in Figure 1. At the end of the treatment, the calcium content was evaluated by and o-cresophtalein complexone method, and expressed as percentage of control. **Panel c**) MSCs cells were treated with 25 nM or 50 nM dasatinib (Das) following the scheme reported in Figure 1. At the end of the treatment, the calcium content was evaluated by alizarin red staining and expressed as percentage of control. Each panel is representative of 5 independent experiments.

The effect of TKI treatment was also evaluated in terms of both AP activity and levels of calcium incorporated in the extracellular matrix. Results showed that dasatinib significantly decreased the AP activity (**Figure 2A**) and, consequently, given the pivotal role of this enzyme in osteogenesis, inhibited the mineralization process (**Figure 2B,2C**).

**Figure 3:**
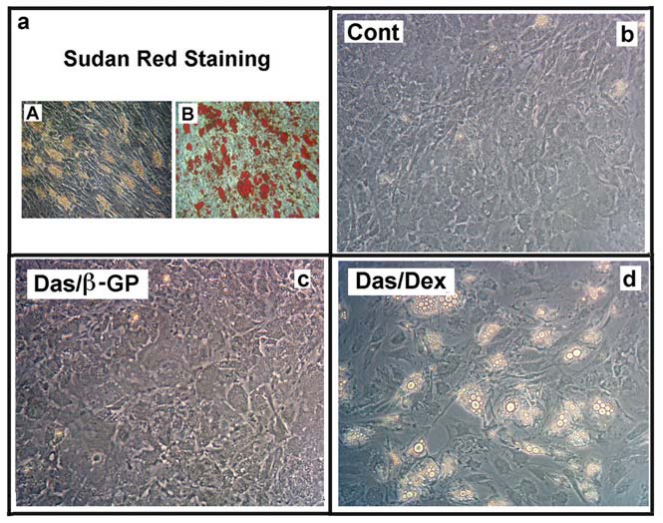
Characterization of dasatinib-dependent MSCs adipogenesis. **Panel a**) MSCs were treated as in Figure 1. At the end of experiments the image of dasatinib-treated cells was captured (**A**) and, then, the cells were stained with the Sudan Red staining method (**B**). **Panels b–d**) MSCs were seeded at a density of 10^4^ cells/cm^2^. After one day, 25 nM dasatinib was added plus β-glycerophosphate (Das/β-GP, **Panel c**) or plus 100 nM dexamethasone (Das/Dex, **Panel d**). Control cells were shown in **Panel b**. Finally, after additional 3 weeks, the images were captured. The panels are representative of 4 independent experiments.

The adipocytic differentiation of dasatinib-treated MSC was also confirmed by Sudan red staining, a method that evidences intracellular lipid vacuole accumulation (**Figure 3A** **)**.

In order to clarify which of the components of the OM could be necessary for inducing adipogenesis, we tested them separately, in the presence of the TKI. As reported in **Figure 3** **(panels B–D)**, only dasatinib plus dexamethasone was able of inducing adipocytic differentiation that did not occur following the treatment with β-glycerophosphate plus the TKI or dexamethasone alone (not shown). Thus, the steroid was the molecule of OM required for the adipogenic effect of dasatinib.

### 2. Dasatinib activates the expression of biochemical markers of adipogenesis in the presence of dexamethasone

The up-regulation of the transcription factor PPARγ (Peroxisome Proliferator-Activated Receptor γ is a key feature of adipocytic differentiation [33], [34]. Thus, we evaluated whether the treatment of MSCs with 25 nM dasatinib followed, after one week, by the addition of 100 nM dexamethasone was able to induce the expression of the transcription factor. After 30 days, the samples were collected and the content of PPARγ was evaluated by semi-quantitative PCR.

**Figure 4:**
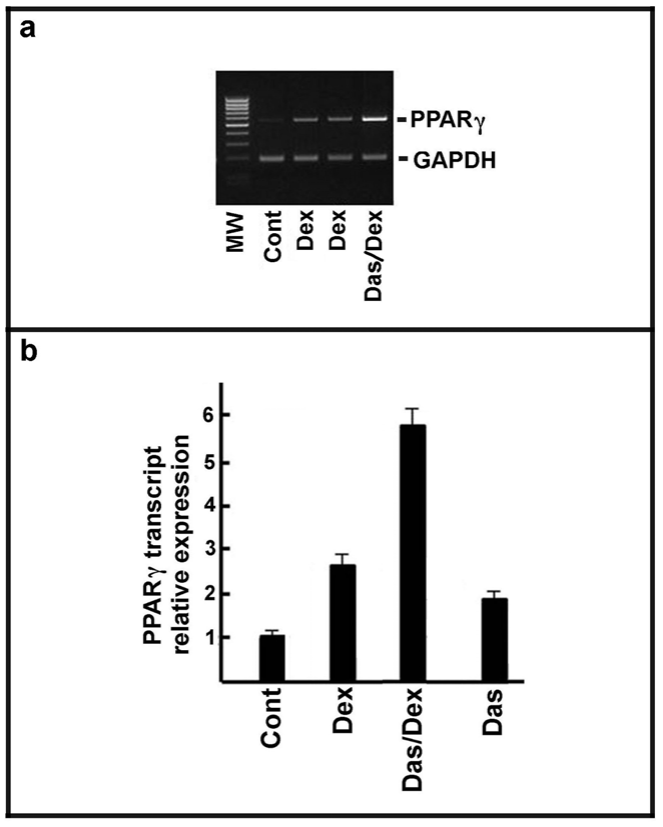
Effect of dasatinib on PPARγ expression in MSCs. **Panel a**) MSCs were seeded at a density of 10^4^ cells/cm^2^. After one day, 25 nM dasatinib (Das) were added. After one week, 100 nM dexamethasone (Dex) was added in some samples as reported in the panel. After further 30 days, total RNA was prepared and the content of PPARγ transcript evaluated by semiquantitative RT-PCR. MW, molecular weight standards. Glyceraldheyde 3-phosphate dehydrogenase (GAPDH) was used as internal standard. Further details of RT-PCR are reported under Materials and Methods Section. **Panel b**) The experiment is similar to that in **Panel 4a** except that the content of PPARγ transcript was determined by quantitative RT-PCR. Further details on this analysis are reported under Materials and Methods Section. The results are expressed as percentage of control. The panels are representative of 5 independent experiments.

As shown in **Figure 4A**, dexamethasone alone faintly increased the expression of the transcription factor, while the addition of dasatinib to dexamethasone strongly up-regulated PPARγ expression. The effect of the compounds on PPARγ transcript level was evaluated by quantitative PCR. As reported in **Figure 4B**, dexamethasone alone increased PPARγ expression of about 2.5 folds, while PPARγ mRNA was strongly enhanced by dasatinib plus dexamethasone (about 6-folds). Intriguingly, dasatinib alone also up-regulated PPARγ mRNA.

In subsequent experiments, we employed another second line BCR-ABL inhibitor, namely bosutinib. This TKI was selected since it is a Src/ABL kinase inhibitor (like dasatinib) with target kinases largely superimposable to those of dasatinib. The employed bosutinib concentration (100 nM) was chosen on the basis of recent pharmacokinetics data [35].

We investigated the effect of dexamethasone alone, or added with 25 nM dasatinib or 100 nM bosutinib. In these experiments, the molecules (i.e dexamethasone and the two TKIs) were added one day after MSCs plating. We also tested the activity of two different concentrations of dexamethasone and the effect of dasatinib alone. After 10 days treatment, total mRNA was recovered and the content of PPARγ CEBPα (CCAAT/enhancer-binding protein alpha), LPL (Lipoprotein Lipase) and SREBP1c (Sterol Regulatory Element-Binding Protein 1c) transcript was evaluated by RT-PCR. CEBPα is a nuclear factor expressed concurrently to PPARγ during the adipocytic differentiation [36], while *LPL* and *SREBP1c* genes are key targets of active PPARγ[37], [38].

**Figure 5:**
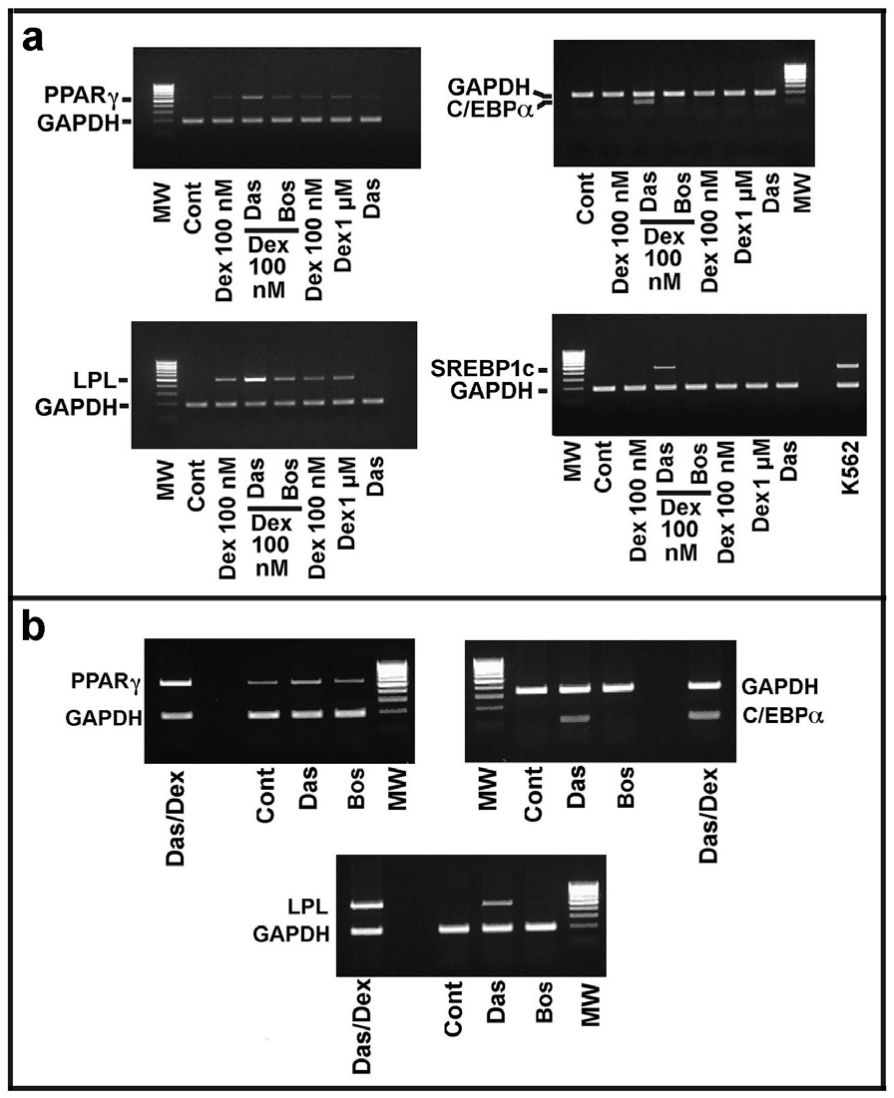
Effect of dasatinib or bosutinib with or without dexamethasone on the expression of PPARγ, CEBPα, LPL and SREBP1c in MSCs. **Panel a**) MSCs were seeded at a density of 10^4^ cells/cm^2^. After one day, the cells were treated with: 100 nM dexamethasone (Dex); 25 nM dasatinib/100nM dexamethasone (Das/Dex); 100 nM bosutinib/100nM dexamethasone (Bos/Dex); 1 µM dexamethasone (Dex); 25 nM dasatinib alone (Das). After 10 days, total RNA was prepared and the content of PPARγ, CEBPα, LPL and SREBP1c was evaluated by semiquantitative RT-PCR. MW, molecular weight standards. Glyceraldheyde 3-phosphate dehydrogenase (GAPDH) was used as internal standard. Furthermore, in the analysis of SREBP1c transcript, a K562 erythroid cell total RNA was employed, since it has been reported that these cells are rich of SREBP1c transcript. Additional details of RT-PCR are reported under Materials and Methods Section. **Panel b**) MSCs were seeded at a density of 10^4^ cells/cm^2^. After one day, the cells were treated respectively with: 25 nM dasatinib/100nM dexamethasone (Das/Dex); 25 nM dasatinib alone (Das), and 100 nM bosutinib (Bos) alone. After 20 days, total RNA was prepared and the content of PPARγ, CEBPα and LPL was evaluated by semiquantitative RT-PCR. MW, molecular weight standards. Glyceraldheyde 3-phosphate dehydrogenase (GAPDH) was used as internal standard. In this experiment, we have increased the number of PCR cycles from 30 to 35 in order to identify the occurrence of low amount of transcript. Additional details of RT-PCR are reported under Materials and Methods Section. The panels are representative of 5 independent experiments.

As reported in **Figure 5A**, only dasatinib/dexamethasone increased the expression of *PPARγ* over the activity of dexamethasone alone. Dasatinib plus dexamethasone was also able to clearly up-regulate the level of *LPL* transcript, thus confirming that the newly formed PPARγ was functionally active. The accumulation of *CEBPα* and *SREBP1c* transcripts was also evident. In this experiment, K562 cell line cDNA was employed as positive control for *SREBP1c* transcript [39].

In order to clarify the effect of dasatinib alone (i.e without the addition of dexamethasone), MSCs were cultured for 20 days in the presence of dasatinib and bosutinib alone. Then, the levels of *PPARγ*, *CEBPα* and *LPL* transcription were evaluated by RT-PCR. In this experiment, the number of PCR cycles was increased (from 30 to 35 cycles) to identify low amounts of transcripts. As shown in **Figure 5B**, dasatinib induced the expression of the three genes investigated, while bosutinib did show any effect. Also in this case, the levels of the transcripts were remarkably increased by the addition of dexamethasone to dasatinib (**Figure 5B**).

### 3. Dasatinib induces a strong increase of nuclear PPARγ protein in MSCs

All together the reported experiments demonstrated that the adipogenic effect of dasatinib on MSCs was observed both at morphological and mRNA level. However, in order to definitely confirm our findings, we investigated PPARγ accumulation also at protein level.

**Figure 6:**
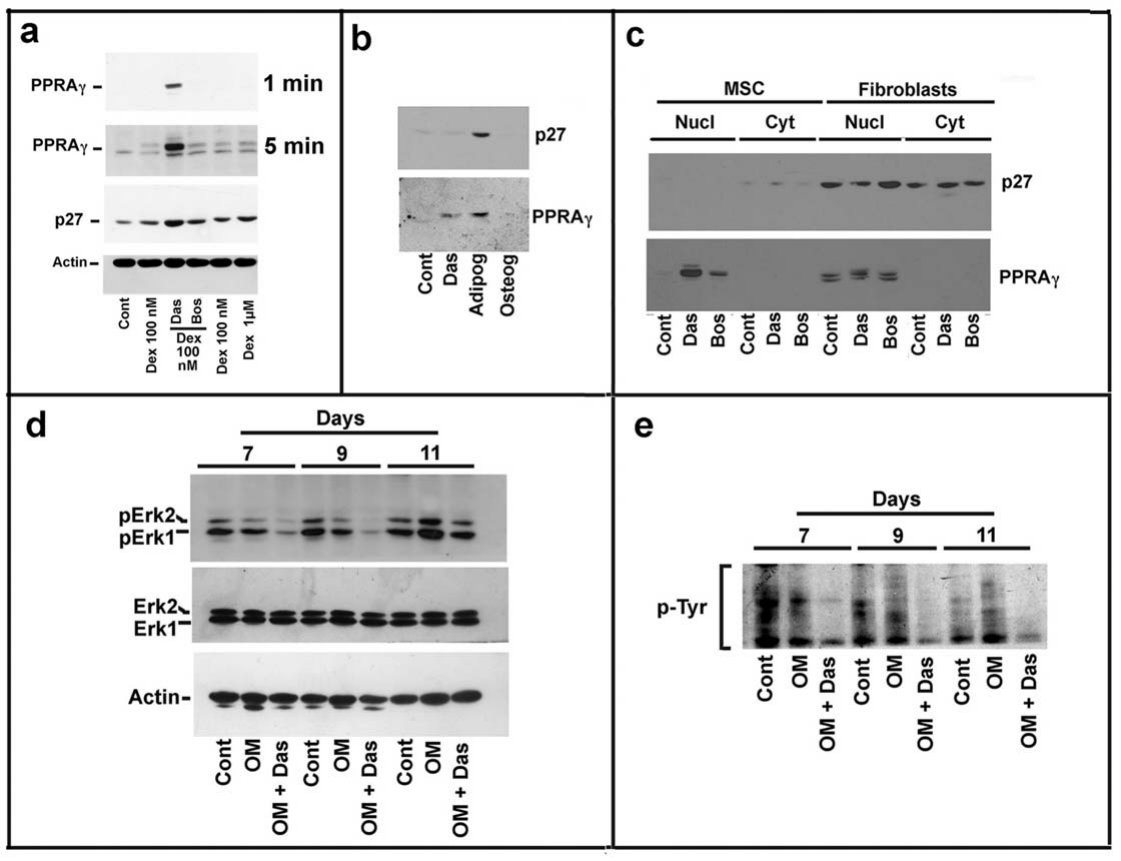
Effect of TKIs with (or without) dexamethasone on the content of PPARγ and p27^Kip1^ proteins in MSCs and primary human fibroblasts. The experiment was performed essentially as reported in Figure 5. **Panel a**) In this case, MSCs total cell extracts were prepared and investigated by immunoblotting for the content of PPARγ and p27^Kip1^ (p27) protein. Actin was used for confirming the equal loading. Two different film expositions (1 and 5 minutes) were performed to obtain a better evaluation of PPARγ protein content. Additional details are reported under Materials and Methods Section. **Panel b**) MSCs were seeded at a density of 10^4^ cells/cm^2^. After one day, MSCs were treated with 25 nM dasatinib (Das) or cultured in adipogenic or osteogenic media. Subsequently, MSCs were cultured for 25 days and then collected. Finally, cell extracts were prepared and investigated by immunoblotting for the content of PPARγ and p27^Kip1^ (p27) protein. **Panel c**) MSCs and human primary skin fibroblasts were seeded at a density of 10^4^ cells/cm^2^. After 24 hours, 25 nM dasatinib (Das) or 100 nM bosutinib (Bos) were added to the cells, After 40 days, the cells were collected and nuclear and cytosolic fractions prepared by Ne-Per as reported in Materials and Methods. The purity of nuclear and cytosolic compartments was evaluated by immunoblotting with anti-HDAC1 (a nuclear protein) and anti-cofillin (a cytolic protein) antibodies. The results confirmed the purity of fractions (data not reported). Finally, the content of PPARγ and p27^Kip1^ (p27) proteins was determined by immunoblotting. **Panels d and e**) MSCs were seeded at a density of of 10^4^ cells/cm^2^. After one day, the medium was removed and substituted with osteogenic medium or osteogenic medium plus dasatinib. At the reported days, MSCs were recovered and employed to evaluate (**Panel d**) phosphoErk1/2 and Erk1/2 levels, or (**Panel e**) the level of phospho-tyrosine protein levels (pY). The panels are representative of 3 independent experiments.

As reported in **Figure 6A**, only dasatinib/dexamethasone increased significantly the level of PPARγ protein. A small increase was also observed with the addition of dexamethasone by itself, but not in the sample treated with bosutinib plus dexamethasone versus dexamethasone alone.

It was also evident that dasatinib plus dexamethasone increased significantly p27^Kip1^, an inhibitor of cyclin-dependent kinases, whose accumulation is both linked to the arrest of cells in G1 phase and, frequently, to the induction of differentiation.

Subsequently, we compared the effect of dasatinib alone with the activity of adipogenic medium and OM on MSCs. In the experiment, that lasted 25 days, we investigated the effect on PPARγand p27^Kip1^ levels. As reported in **Figure 6B**, the adipogenic medium caused a clear increase of PPARγ and dasatinib by itself induced a small increase of the transcription factor. Conversely, in both untreated and OM treated MSCs no accumulation of PPARγ was observed. The adipogenic medium also caused a strong up-regulation of p27^Kip1^, similarly to the effect shown in **Figure 6A** by dasatinib plus dexamethasone.

In **Figure 6C** **,** we report the effect of dasatinib and bosutinib alone after a period of incubation of 40 days. In this experiment, primary cultures of human skin fibroblasts (a different mesenchymal-derived cell phenotype) were also employed. Both the nuclear and the cytosolic compartments were analyzed in order to investigate PPARγ localization. Dasatinib alone significantly increased the level of the nuclear transcription factor in MSCs. A slight increase was also observed in bosutinib treated MSCs. Intriguingly, PPARγ was localized exclusively at nuclear level, indirectly confirming its activity. The level of PPARγ did not change in human fibroblasts before and after the treatment with dasatinib and bosutinib, thus indicating that MSCs are specific targets of the TKI actcivity. Interestingly, a faint but clear increase of p27^Kip1^ was observed in the cytosol of dasatinib-treated MSCs.

### 4. Dasatinib inhibits the activity of MAPK pathway in MSCs

It has been previously reported that the treatment with the OM increased in MSCs, at specific time interval, the activity of the MAPK pathway [40]. Accordingly, MAPK inhibitors reduced the osteogenic differentiation and induced adipogenesis [40]. Finally, the treatment with the adipogenic medium significantly reduced the activity of basal MAPK activity [40]. These findings suggest that human MSCs osteogenesis is associated to activation of MAPK pathway while down-regulation of Erk1/2 activation might lead to MSCs adipogenesis [40], [41]. Since dasatinib is a strong inhibitor of several intracellular pathways including MAPK [14], we hypothesized that its adipogenic activity might in part be attributable to an effect on the signal transduction. Thus, we evaluated the activity of the TKI on Erk1/2 phosphorylation. As shown in **Figure 6D**, the molecule significantly reduced the degree of MAPK pathway activity (**days 7 and 9**) and prevented its activation by the OM (**day 11**). These findings suggest that the down-regulation of MAPK pathway might be involved in the effect of dasatinib on MSCs. The activity of dasatinib as tyrosine kinase inhibitor in this experiment was confirmed by re-analyzing the filter employed in **Figure 6D** with antibodies against phosphotyrosine. A clear decrease of tyrosine phosphorylation pattern was observed in MSCs treated with molecule (**Figure 6E**).

## Discussion

In this study, we report for the first time that dasatinib, at concentrations equivalent to those reached in serum during CML treatment, induces the adipocytic differentiation of MSCs. The heretofore unreported effect is endowed with significant clinical relevance, being now the therapy of CML with TKIs a long-lasting treatment. Importantly, dasatinib is also under evaluation for the therapy of several solid cancers, including the carcinoma of pancreas, prostate, breast, ovary and colon, as well as non-small cell lung carcinoma [42]–[55].

The dasatinib-dependent MSCs adipocytic differentiation is characterized by an initial accumulation of small lipid droplets that fused in part, becoming larger cytosolic vacuoles. The positivity to Sudan Red staining confirmed their lipidic content. We also demonstrated that dasatinib-dependent adipogenesis requires dexamethasone at a concentration (100 nM) that alone does not affect MSCs phenotype.

The effect of dasatinib on MSCs was confirmed by a large series of molecular evidence. The TKI plus the corticosteroid strongly induced the nuclear accumulation of PPARγ, a transcription factor that not only is critical for adipogenesis, but also represents a specific marker of fat cell differentiation. Up-regulated PPARγ protein was fully active, since its increase stimulated the expression of *LPL* and *CREBP1c* genes, two of its specific targets [33]–[37]. The dasatinib-dependent MSCs adipogenesis was further confirmed by the accumulation of CEBPα transcript, an independent marker of adipocytic differentiation.

While the observation that dasatinib plus dexamethasone induces adipogenesis is sustained by several morphological and molecular data, the effects of dasatinib alone on MSCs phenotype seem more complex to characterize. Several data (**Figures 4B** **,** **5B** **and** **6B**) suggest that the TKI induces PPARγ up-regulation, although at a much minor extent than when it was associated to dexamethasone. However, under specific RT-PCR conditions (i.e. a lower number of cycles), the effect of the TKI on PPARγ level was not evident (**Figure 5A**). The difference might also be explained with the length of treatment (10 versus 20 days) being, probably, the effect of dasatinib alone evident only after a longer incubation period.

Adipogenic commitment of MSCs can be obtained *in vitro* by the addition of a medium containing dexamethasone (1 µM), insulin and 3-isobutyl-1-methylxanthine (a cAMP phosphodiesterase inhibitor). However, dexamethasone alone at 100 nM does not induce adipogenic differentiation (not shown).

Dasatinib is a powerful inhibitor of several Src and Src-like kinases, and, thus it can affect a plethora of cellular events and protein-protein interactions, including transduction pathways correlated to cell lineage. In order to investigate potential molecular mechanisms involved in MSCs adipogenesis and affected by dasatinib, we pointed our attention on the MAPK transduction pathway. As a matter of a fact, several studies demonstrated that inhibition of Erk1/2 activity might influence MSCs differentiation. Particularly, it has been reported that OM induces the activation of Erk1/2 kinases during the process of MSCs osteoblastic differentiation while the adipogenic medium reduces remarkably Erk1/2 phosphorylation. Literature data also demonstrated that MSCs treatment with inhibitors of MAPK pathway induces adipogenesis. Therefore, the inhibition of Erk1/2 phosphorylation might be an interesting molecular mechanism responsible for the dasatinib-dependent MSCs adipogenesis. As reported in **Figure 6D**, dasatinib clearly inhibits Erk1/2 activity at 7 and 9 days of treatment and strongly reduces, at 11 days, its activation by OM, thus suggesting that this dasatinib effect on MAPK inhibition might have a role in the commitment of MSCs towards adipogenesis.

The effect of dexamethasone might be also taken into consideration. Indeed, our experiments demonstrate that the molecule by itself might up-regulate PPARγ expression, although at a level not sufficient to cause morphological differentiation. On the other hand, 1 µM dexamethasone is a component of the adipogenic medium, suggesting its importance in the adipocytic commitment. It is probable that the dasatinib-associated inhibition of Erk1/2 activity might induce a partial increase of PPARγ strongly potentiated by dexamethsone.

During the development of this project, studies describing the effect of dasatinib on human MSCs osteogenesis (but not its adipogenic activity) have been published [56]–[58]. However, the reported findings significantly disagreed [56]–[58]. Jonsson and colleagues showed that dasatinib inhibits the proliferation of MSC and their bone morphogenetic protein-dependent osteoblastic differentiation [56]. This inhibition was demonstrated by reduction of AP activity, diminished calcification and reduced levels of Runx2 and Osterix transcripts (two markers of osteoblastic differentiation) [56]. The effect was evidenced at dasatinib therapeutic concentrations (5**–**80 nM) [56].

Conversely, two studies suggested that dasatinib accelerates the MSCs osteoblastic differentiation [57], [58]. In one report, a significant increase of AP activity and calcium deposition was observed in MSCs when the molecule was added in association with β-glycerophosphate after, respectively, 7 and 14 days [57]. In the other investigation, MSCs were induced to differentiate in osteoblastic cells by treatment with OM with or without dasatinib [58]. The authors also found that the addition of dasatinib induced expression of mRNAs of osteogenic markers and accelerated mineralization, as compared with cultures grown in standard medium or OM only [58].

Our study confirms that MCSs treatment with dasatinib prevents osteogenesis [56]. We have also observed, according to Jonsson and colleagues [56] and to experiments on imatinib [59] that BCR-ABL inhibitors reduce MSCs proliferation (data not showed). However, the major novelty of our investigation is the observation, described for the first time, that dasatinib induces adipogenic differentiation at a concentration similar to that occurring *in vivo*. Moreover, the inhibition of Erk1/2 activity, a well known effect of Src inhibitors, might direct (at least in part) MSCs towards adipogenesis, in accordance with several previous investigations [40], [41]. Finally, it is reasonable that the inhibition of osteoblastic differentiation by dasatinib might drive the cells towards the adipogenesis, particularly in the presence of dexamethasone.

The adipogenic effect of dasatinib on human MSCs is of peculiar clinical interest. Indeed, the molecule represents a pivotal drug in the treatment of CML. Since this neoplasia must be considered a chronic disease, changes of the mesenchymal component of bone marrow (after a prolonged therapy) might represent an important potential side event that can affect both bone turnover and hematopoiesis. The observation might have even more relevance in view of the proposed use of the Src inhibitor in the therapy of several solid cancers. Finally, our findings suggest that strong caution must be taken in considering, among the already clinically identified dasatinib side effects, the possibility that it can cause a pathological adipogenesis of bone marrow mesenchymal component.

## Supporting Information

Figure S1 **Immunophenotype of mesenchymal stem cells from human bone marrow.** MSC cells were prepared as reported in Materials and Methods. MSCs from passage 2 were harvested and labeled with antibodies against CD105 and CD29 (positive MSCs markers) and CD45, CD34 and CD14 (MSCs negative markers) and analyzed by FACS. Histograms represent the staining of cells with the indicated antibody.
(TIF)
