# Supplementary figures and images for: The Tyrosine Kinase Inhibitor Dasatinib Induces a Marked Adipogenic Differentiation of Human Multipotent Mesenchymal Stromal Cells
Source: PLoS One. 2011 Dec 2;6(12):e28555. doi: 10.1371/journal.pone.0028555 (PMC3229607; doi:10.1371/journal.pone.0028555)

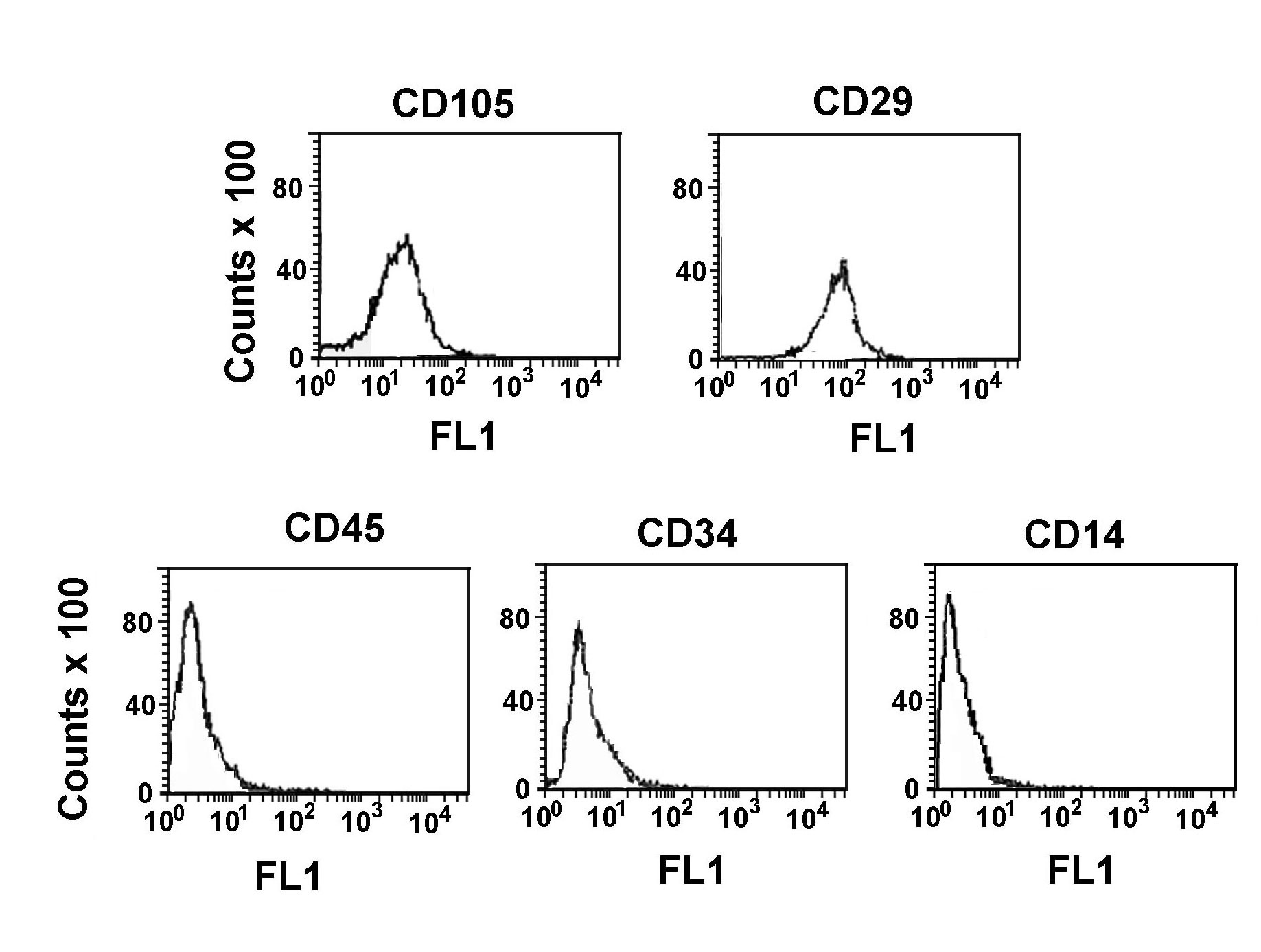

Supplement: Figure S1 — Immunophenotype of mesenchymal stem cells from human bone marrow. MSC cells were prepared as reported in Materials and Methods. MSCs from passage 2 were harvested and labeled with antibodies against CD105 and CD29 (positive MSCs markers) and CD45, CD34 and CD14 (MSCs negative markers) and analyzed by FACS. Histograms represent the staining of cells with the indicated antibody. (TIF) [file pone.0028555.s001.tif]
